# Applying team strategies for dynamic coordination: A comparative study of expertise using 3-on-3 basketball

**DOI:** 10.1371/journal.pone.0343077

**Published:** 2026-02-20

**Authors:** Jun Ichikawa, Masatoshi Yamada, Yutaka Iwaihara, Genki Ichinose, Keisuke Fujii

**Affiliations:** 1 Faculty of Informatics, Shizuoka University, 3-5-1 Johoku, Chuo-ku, Hamamatsu, Shizuoka, Japan; 2 Faculty of Business Administration, Tokoha University, 6-1 Yayoi-cho, Suruga-ku, Shizuoka, Shizuoka, Japan; 3 Faculty of Global Interdisciplinary Science and Innovation, Shizuoka University, 836 Ohya, Suruga-ku, Shizuoka, Shizuoka, Japan,; 4 Faculty of Engineering, Shizuoka University, 3-5-1 Johoku, Chuo-ku, Hamamatsu, Shizuoka, Japan; 5 Graduate School of Informatics, Nagoya University, Furo-cho, Chikusa-ku, Nagoya, Aichi, Japan; 6 RIKEN Center for Advanced Intelligence Project, 1-5 Yamadaoka, Suita, Osaka, Japan; Universidade Federal de Goias, BRAZIL

## Abstract

Humans working as a team can achieve higher performance. Studies in sports science, network science, and machine learning have extracted dynamic physical interaction structures of such coordination in team sports. However, the information processing, such as the application of team strategies, has not been fully discussed. The purpose of this cognitive science study was to investigate the application of team strategies for dynamic coordination across different expertise levels using 3-on-3 basketball. In a field experiment, female players, who were selected as prefecture representatives in Japan (Average experience 19.33 years, SD = 4.09), repeatedly engaged in mini-games. Their previous and current affiliated teams competed in national tournaments. We analyzed the difficulty in anticipating offensive movements for the opponent defensive team, quantified as entropy, using tracking position data. This was compared with female players recorded in a previous study. They affiliated with a university team ranked in the third division of the regional league (Average experience 11.33 years, SD = 3.42). There was no such achievement as those in the high expertise condition. Using a linear mixed model with the significance level (α=0.05), the results showed that the entropy for the key player in the high expertise condition was significantly higher than that in the low expertise after the tips condition, and similar to that in the low expertise before the tips condition. The tips were concise coaching advice regarding coordination related to the crucial role of intervention decision and adjustment. This was also lower than that simulated in the random walk condition, which served as a minimal baseline for scientifically explaining the observed complexity. Our first step study suggests that the movement dynamics at the expert level may be relatively complex, making it difficult for the defensive team to anticipate, and related to the application of team strategies.

## Introduction

Humans working as a team can achieve higher performance. In various activities, such as business projects, orchestras, and outdoor cooking, members share roles; the vast knowledge and behaviors are distributed. The members share or complement crucial information through role-sharing interactions [1–3], consolidate it, and establish a socio-cognitive system [4–6]. This helps reduce the workload for each individual. In this study, we focus on planned coordination [7], defined as role-sharing interactions that enable a team to achieve a common goal and higher performance. Many research fields have investigated efficient and effective coordination. The discussions mentioned above are consistent with theories of distributed cognition and collective intelligence [8–10]. Team sports are a representative example of coordination based on the socio-cognitive system. A team must apply strategies within limited rules, time, and play space to achieve the common goal of winning against the opponent. Thus, team sports can be regarded as micro-models of society and provide valuable insight into understanding of real-world coordination. This cognitive science study investigates information processing regarding the application of team strategies using team sports.

Coordination among multiple players observed in team sports diversifies relationships between roles and dynamically evolves through primitive movements [11,12]. In related work, sports science, network science, and machine learning studies have investigated adaptive and flexible coordination by expert teams. The interactions evolve depending on the situation and team strategies [13,14]. These data-driven approaches have extracted mechanistic and geometrical structures of physical interactions, and predict performance and ideal behaviors (e.g., [15–17]). Then, tracking data have been often used, which record time-series players’ action events (e.g., dribbling and passing) and their positions. Studies in sports science and network science have analyzed behavioral synchrony in relation to smoothly applying team strategies, as well as reasonable areas in which an offensive player can pass the ball or a defensive player can prevent the pass. Mathematical and geometrical frameworks have been used [18–20]. Furthermore, passing networks, in which players are nodes and relationships between passing and receiving are links, have been established to examine variations in team formations, specific passing patterns, crucial players, and robustness [21–24]. To evaluate team strategies, studies in machine learning have developed techniques for estimating the probabilities of action event occurrences, such as shooting and ball possessions [25,26], as well as for predicting players’ time-series trajectories [27]. In addition, a counterfactual simulation method has been introduced to evaluate decisions whether and when a player should pass the ball [28]. As a notable network science research, Bourgeais et al. examined offensive coordination using tracking data of passing sequences in professional basketball [29]. They calculated entropy, based on Shannon’s information theory [30], and evaluated movement dynamics. The results suggested that high entropy represents complexity with the difficulty in anticipating movements for the opponent; it is related to the diversity of coordination structures and high team performance. Meanwhile, regardless of team sports, cognitive science studies have emphasized the importance of a shared mental model that underlies coordination. Such a model is a representation based on structured knowledge and strategies as a common ground in a team [31–33]. This is established by sharing or complementing information according to relationships between roles, enabling efficient activities [34–36]. It allows a team to estimate future situations, make plans, and explain and anticipate each other’s behaviors [37–39]. The team can apply strategies without verbal communication [40–42].

Although these findings provide insight into understanding of coordination processes, few studies have investigated the application of team strategies, which serves as the foundation for dynamic behaviors. Cognitive science has often examined the factors of information processing, which influence on team performance by manipulating experimental tasks and environments. However, real-world coordination, such as team sports, has not been fully investigated because of difficulties in experimental control [12]. Sports science, network science, and machine learning have investigated the physical structures and dynamics in team sports, in which roles switch and overlap [43]. These studies have focused on the behaviors themselves, while the information processing has remained outside their scope [11,12,44]. At the expert level in team sports, the application of diverse strategies through enriched shared mental model would be reflected in movement dynamics. Its characteristics may be related to complexity with the difficulty in anticipating movements.

Our approach aimed to bridge the gap between the quantitative analysis of movement dynamics and the information processing. The purpose of this study was to investigate the application of team strategies for dynamic coordination across different expertise levels using 3-on-3 basketball. According to the findings introduced earlier, if the application of several team strategies is related to movement dynamics, then (1) the movement may be complex, making it difficult for the opponent to anticipate. Naturally, (2) its characteristics are not artificial random movement at the minimum baseline level. To investigate point (1), we focused on offensive coordination and conducted a field experiment under the high expertise condition, in which female players were selected as the prefecture representatives in Japan. Their dynamic behaviors were recorded and tracking position data were collected. We calculated entropy to evaluate the difficulty in anticipating offensive movements for the opponent defensive team. This study compared it with those in two conditions: the low expertise before the tips condition and the low expertise after the tips condition in the previous study [12]. In the low expertise condition, female players were affiliated with the university team ranked in the third division of the regional league. The tips were concise coaching advice regarding coordination related to the key role of intervention decision and adjustment. Consequently, the offensive team established a representative coordination pattern by the tips. The low expertise before the tips condition might not establish the particular pattern, compared with the low expertise after the tips condition (see the Comparisons with the lower expertise team section). Furthermore, to investigate point (2), we included an additional random walk condition in which agents’ movements were simulated.

Therefore, we formulated the hypotheses; H1 and H2 correspond to points (1) and (2). These characteristics are expected to be particularly observed in the key role of intervention decision and adjustment. H1 consists of two different perspectives.

- H1-1: In the high expertise condition, the entropy representing the difficulty in anticipating the offensive movement is higher than that in the low expertise after the tips condition.
- H1-2: In the high expertise condition, the entropy is similar to that in the low expertise before the tips condition.
- H2: In the high expertise condition, the entropy is lower than that in the random walk condition.

As a reference, this study also conducted an additional fundamental analysis to supplement (H1-2). We calculated the coefficient of variation (CV), which represents the variability in the distance between the offensive player in the key role and each other player. Typical offensive coordination, such as “hand-off” and “pick and roll”, may be related to a relatively short distance between the key player and each teammate. In contrast, another coordination, such as the key player running to a corner area and attempting a three-point shot, may be related to a relatively large distance between the key player and each teammate. In addition, if the CV in the distance between the key player and each defensive player is large, it may be related to unstable or disrupted defense. Thus, the application of team strategies may be related to the CV, as described in (H3) below.

- H3: In the high expertise condition, the CV is larger than that in the low expertise before the tips condition.

In the following sections, we carefully explained the details of the field experiment and data analysis to test the hypotheses.

## Methods

### Participants

Six female regular players participated in the 3-on-3 basketball field experiment. They were selected as Shizuoka Prefecture representatives in Japan; the players were higher expertise. The participants regularly played 5-on-5 basketball, and their averages of age, height, and experience were 24.67 years old (SD=3.20), 168.83 cm (SD=7.56), and 17.33 years (SD=4.06), respectively. The head coach (the third author), who thoroughly understood the players through regular practice and games, divided them into the offensive and defensive teams ensuring their abilities were competitive based on the profile data. It included handedness and current and previous positions (see the Comparisons with the lower expertise team section). The team assignment was fixed throughout this field experiment to avoid the influence on performance and movements.

### Informed consent

The research project was approved and noticed on December 21, 2023, by the Ethics and Safety Committee of Shizuoka University (Approval No. 23–58), with which the first author was affiliated and responsible for data collection, storage, and analysis. From June 25 to July 12, 2024, the first author requested participants via the third author. We explained how we video-recorded and collected the data, and written informed consent was obtained from all the players. According to the informed consent, the image in the manuscript does not include individual identifiers.

### Designed 3-on-3 basketball game

**Fig 1:**
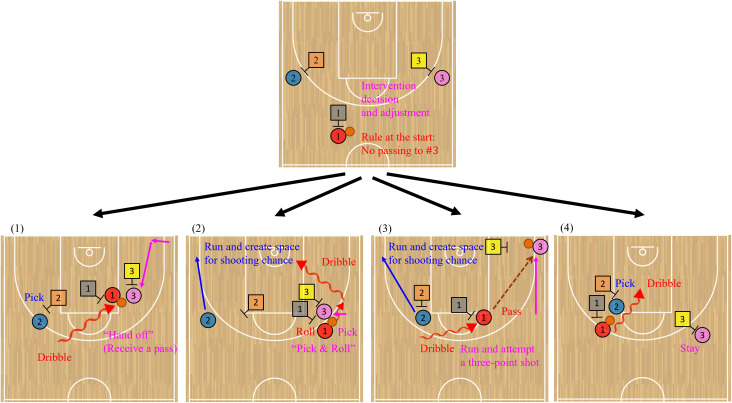
Diagrams of 3-on-3 basketball mini-game in the field experiment to examine offensive coordination. These illustrations are redesigned in the previous study [12] based on CC-BY. The original explanations are referred from the previous study. Circles and squares represent offensive and defensive players, respectively. The initial position of each offensive player is instructed in order to maintain certain distances among the players by the main experimenter. The defensive players (#1–#3) match up against the offensive players (#1–#3). The defensive players’ initial positions are self-selected. Just before each game, the experimenter instructs the two-man game position randomly on the right or left side of the goal. The ball handler is always offensive #1, and each game starts without direct passing to offensive #3. To win the mini-game within a 15 s time limit, offensive #3 should implement the key role of intervention decision and adjustment. This player needs to intervene with the teammates and adjust the two-man game to create favorable situations, such as (1) “hand-off,” (2) “pick and roll,” and (3) running to a corner area to attempt a three-point shot. Furthermore, it is crucial for offensive #3 (4) to stay in place without interrupting the teammates to ensure their play space. Solid arrows represent the movements of the off-ball player, zigzag arrows present those of the on-ball player, and a dotted arrow indicates the pass trajectory.

Fig 1 illustrates a diagram of the mini-game in the field experiment. This game was designed to examine offensive coordination (see the original explanations in the previous study [12] under CC-BY). In the diagram, the circles and squares represent offensive and defensive players, respectively.

The win condition for the offensive team is to create open space for an unmarked shooting chance by an offensive player drawing a defensive player within a 15-s time limit (see the Procedures for offensive team performance section). In contrast, the defensive team must prevent the criterion. The purpose of the current and previous studies was to investigate the coordination processes involving off-ball players, and the criteria prioritized role-sharing interactions leading up to the shot. In the mini-game, several rules are prepared to ensure consistent starting situations. The initial position of each offensive player is determined in order to maintain certain distances among the players. The defensive players (#1–#3) are instructed to match up against the offensive players (#1–#3). The defensive players’ initial positions are self-selected. The positions of offensive #1 and offensive #2 are designed as “guard” and “wing”, which are typical configurations in 5-on-5 basketball. Just before each game, the two-man game position is randomly on the right or left side of the goal, as shown on the right side in Fig 1, in order to control the influence of the dominant hand. The ball handler is always offensive #1, and each game starts without direct passing to offensive #3. These rules are set to observe the coordination processes involving offensive #3 in a key role described below. At this time, this field experiment was allowed to continue the game after a rebound. Meanwhile, under the standard shot-clock duration in 3-on-3 basketball (12 s), actual playing tends to become extremely fast, which may lead the offensive team to rely more on individual skills rather than coordination. Thus, we set the time limit slightly longer than 12 s and adopted a 15-s duration. However, we cannot fully exclude the possibility that this time limit had both negative psychological and physical influences on the players. This point warrants further examination in future studies.

To win the mini-game, offensive #3 should implement the key role of intervention decision and adjustment. This player needs to intervene with the teammates, namely offensive #1, who is the role of primarily attacking by dribbling toward the goal, and offensive #2, who is the role of cooperating with offensive #1 to create shooting chances. Offensive #3 adjusts the two-man game to create favorable situations. Fig 1 also illustrates the details of coordination, such as (1) “hand-off,” (2) “pick and roll,” and (3) running to a corner area (“deep corner”) to attempt a three-point shot. “Hand-off” involves approaching the on-ball player and receiving a pass directly. “Pick and roll” involves setting a screen (pick) against a defensive player, allowing a teammate to use the screen, evade the defense, and dribble toward the goal (roll). Furthermore, it is crucial for offensive #3 (4) to stay in place without interrupting the teammates to ensure their play space. These coordination are also fundamental in 5-on-5 basketball.

### Experimental environment and procedures

**Fig 2:**
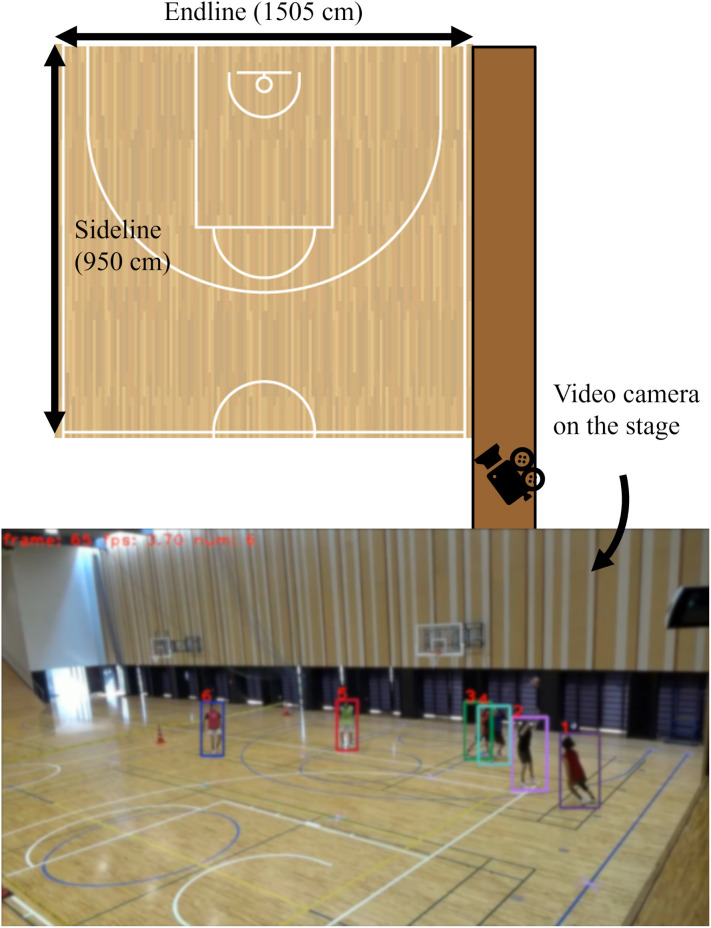
Environment in the field experiment. The upper part is redesigned from the figure in the previous study [12] based on CC-BY. The court area in the university gymnasium conforms to the official size of a 3-on-3 basketball court [45], except for the sideline. The video camera is set on the stage and covered the entire area and all the players from a bird’s-eye view. They are tracked using the image processing algorithm ByteTrack [48]. Written informed consent was obtained from all the players. According to this consent, the image is shown while blurring to avoid including individual identifiers.

Fig 2 illustrates the environment of the field experiment. The court area in the university gymnasium conformed to the official size of a 3-on-3 basketball court [45], except for the sideline. A digital countdown timer was utilized to display the remaining time, and a video camera (Sony Corp., HDR-CX680) was used to record the mini-games. The camera was set on the stage and covered the entire area and all the players from a bird’s-eye view, as shown in Fig 2.

For the experimental procedures, after the players gathered at the venue, the main experimenter (the first author) explained the overall schedule (S1 Note). The players did warm-up exercises, were divided into the offensive and defensive teams, and were given a color-coded bib for individual identification, as illustrated in Fig 1. The experimenter additionally explained the mini-game’s rules. Following this, two practice trials were conducted so that the players could confirm the rules. After that, three sessions comprising seven trials per session were conducted, yielding a total of 21 trials. The interval between trials was approximately 30 s, and the interval between sessions was about one min. Before restarting each session, the experimenter checked the players for signs of fatigue to eliminate this influence on performance and movements.

## Analysis

### Comparisons with the lower expertise team

The experimental environment and procedures, including the devices, were identical to those in the previous study [12]. We conducted a cross-sectional comparison of offensive movements with those in the previous study. Table 1 summarizes the profile survey for the female players who participated in the current and previous studies, respectively. To avoid identifying the individuals, the aggregated data for the offensive and defensive teams are indicated. Notable differences were confirmed between the high and low expertise conditions, particularly in age and experience. However, in Japan basketball, both conditions fall within the same age category. Furthermore, as evidence of achievement, the players in the high expertise condition were selected as Shizuoka Prefecture representatives, and the affiliations of five out of the six players competed in national tournaments. In the low expertise condition, the players affiliated with the university team ranked in the third division of the Tokai area league. They were not selected as the prefecture representatives, and no such achievement in the high expertise condition was recorded.

**Table 1:** Profiles of the offensive and defensive teams in the high and low expertise conditions.

Condition	Team	Age (years old)	Height (cm)	Experience (years)	Dominant hand
High expertise	Offense	26.00 SD=3.74	170.67 SD=8.02	19.33 SD=4.09	All the female players are right-handed.
	Defense	23.33 SD=1.70	167.00 SD=6.57	15.33 SD=2.87	
Low expertise	Offense	19.33 SD=0.94	162.50 SD=5.31	11.33 SD=3.42	All the female players are right-handed.
	Defense	19.00 SD=0.82	167.67 SD=5.25	9.83 SD=1.31	
Condition	Team	Current position		Previous position	
High expertise	Offense	shooting guard, power forward, and center		point guard, power forward, center, shooting guard, and small forward	
	Defense	point guard, small forward, and center		point guard, small forward, center, shooting guard, and power forward	
Low expertise	Offense	point guard, small forward, power forward, and center		point guard, shooting guard, small forward, power forward, and center	
	Defense	shooting guard, small forward, and center		point guard, shooting guard, small forward, power forward, and center	

In the low expertise condition, the field experiment was conducted to practically apply the findings regarding the key role of intervention decision and adjustment in coordination, obtained using the experimental task [44]. The six players were divided into offensive and defensive teams and repeatedly engaged in the mini-games. A pretest was conducted consisting of three sessions, yielding a total of 21 trials. After that, a guest coach gave concise advice regarding coordination related to the key role to the offensive team once. The tips were comprehensive explanations for acquiring the team strategy based on the observations of the pretest, as shown in Fig 1. These details included that when defensive #3 helps, offensive #3 should move to where the driving teammate can see offensive #3, such as a corner area (“deep corner”). Offensive #3 can attempt a three-point shot in the created open space. A following exception was also included: if offensive #3 is in a high position along the three-point line, defensive #3’s back is visible from offensive #3, and defensive #3 goes for help, offensive #3 should dive under the goal. This is because moving along the three-point line and receiving the pass take time. In addition, it was advised that offensive #3 should not move too much and carefully check the defensive players’ positions while staying in place. The summary volume was approximately 230 words, and the advice duration lasted about 7 min 30 s. Following this, a posttest consisting of three sessions was conducted to examine the effect of the tips. The abstract results suggested that the offensive team established a representative coordination pattern by the tips, such as the key player running to a corner area and attempting a three-point shot. This coordination might be related to temporal high team performance (see the Results of offensive team performance section). Before the tips, such a pattern might not be clearly observed.

A limitation was that the players in both conditions were drawn from only a single affiliation, respectively. Even if statistical analysis that considers their profile data is conducted, it is difficult to fully ensure methodological rigor. The issue of sample size is crucial for enhancing the validity and generalizability of the findings as future work.

### Procedures for offensive team performance

This study counted the number of wins in the mini-games for the offensive team in the high expertise condition as team performance. On the day of the field experiment, the head coach of the players (the third author) served as the referee, and all the trials were judged in real time. Subsequently, the second author, who was one of the experimenters, checked the video recordings of all the trials and evaluated the offensive team performance separately from that of the third author. We calculated Cohen’s Kappa coefficient to confirm the degree of matching between the two evaluators. For the trials in which the initial judgments did not match, the third and fifth authors checked the video recordings for the first time and evaluated them independently. The fifth author did not participate in the field experiment. Ultimately, these wins or losses were determined by the majority vote, including that of the second author.

**Fig 3:**
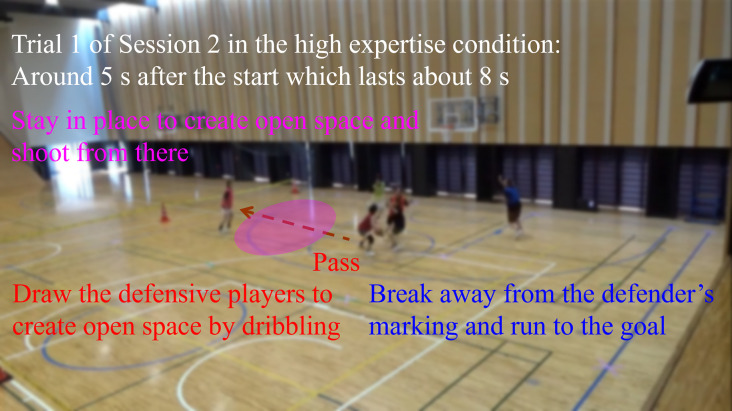
A typical example of judgment in the mini-game. In Trial 1 of Session 2, offensive #3 wearing a pink bib took a three-point shot, and the two evaluators on the first step judged a win for the offensive team. A dotted arrow indicates the pass trajectory. Written informed consent was obtained from all the players. According to this consent, the image is shown while blurring to avoid including individual identifiers.

The second and third authors served as the head coaches of the women’s basketball teams at their affiliated universities. They held coaching licenses certified from the Japan Basketball Association, and had records of leading university teams to national tournaments. The fifth author also served as a coach for the men’s basketball team at the university during his graduate school, and has currently published research articles on basketball [28,43]. Each evaluator judged the offensive team performance based on the following criteria; Fig 3 shows a typical example. When the on-ball player drew the defensive players and the off-ball player stayed in place, open space was created for an unmarked shooting chance; the trial was judged as a win for the offensive team. This is a fundamental team strategy in basketball regardless of 3-on-3 [46,47]. These criteria were that experts could reliably evaluate the mini-game. Even if a careless mistake occurred at the end of the trial or the shot was unsuccessful, the trial was judged as a win when appropriate positioning was achieved just before the shot. In contrast, if a shot was successful made from inappropriate positioning by chance, the trial was judged as a loss. The purpose of this study was to investigate the coordination processes involving off-ball players, and the criteria prioritized role-sharing interactions leading up to the shot. The mini-game in the low expertise condition was evaluated in the previous study in the same manner [12].

However, because of limitations in the image processing technique and the experimental environment, the ball could not be tracked from the video recordings. Furthermore, only a few actions, such as passing and catching, were observed because the mini-game was limited within a 15-s duration in principle. It was difficult to automatically determine the offensive team performance by combining these action events with the position data. Developing an objective evaluation method that does not depend on experts remains an issue for future research.

### Procedures for offensive movements making it difficult for the defensive team to anticipate

To verify hypotheses (H1-1), (H1-2), and (H2) explained in the Introduction section, this study analyzed the offensive movements in the high expertise, low expertise before the tips, and low expertise after the tips, and random walk conditions.

Similar to the previous study [12], all the six players in the recordings (20 fps, 1,280 px × 720 px) were tracked using the image processing algorithm ByteTrack [48]. The experimenter manually corrected missed and undetected errors using the labeling tool Labelbox [49], and generated a two-dimensional dataset from real coordinate transformations. The average of absolute measurement errors was 2.390 cm × 1.557 cm, which was comparable to the errors reported in previous studies on basketball and soccer [12,50], indicating no major issues for analysis.

**Fig 4:**
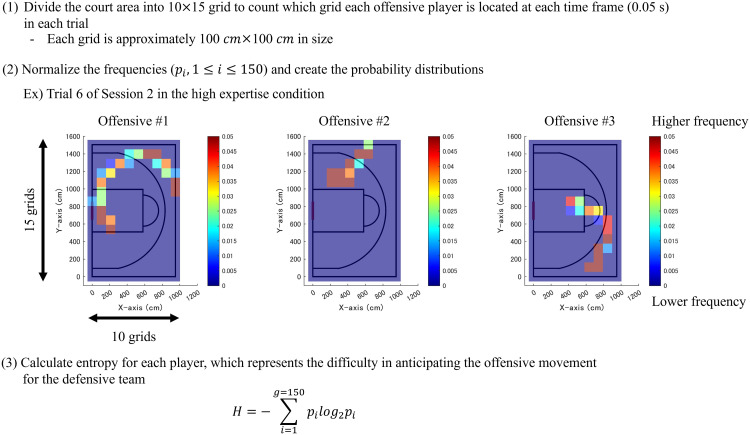
Analysis procedures of entropy representing the difficulty in anticipating offensive movements for the defensive team. A higher entropy 𝐇 indicates that the movement is more complex with higher uncertainty, making it difficult to anticipate.


Using the time-series position data, this analysis calculated entropy for each offensive player, which is defined as an index of uncertainty and unlikelihood of occurrence. A higher entropy value is greater averaged uncertainty and a larger amount of information. In this study, it was interpreted that the movement was diverse and more difficult for the defensive team to anticipate. Fig 4 shows the details; this concept and procedures were referred from the previous study [29]. In each trial, this study divided the court into grids along the sideline (x-direction) and endline (y-direction), and counted where each offensive player was located at each time frame (0.05 s). The frequencies were normalized to create the probability distributions, as shown by the heatmaps in Fig 4, and entropy H was calculated for each player using Equation (1):

$$H=-\sum {\mathrm{\backslash nolimits}}_{i=\text{1}}^{g=\text{1}\text{5}\text{0}}p_{i}{log}_{\text{2}}p_{i}\;.\;$$ (1)

g represents the total number of grids (150), i denotes each grid number (1≤i≤150), and $p_{i}$ indicates the normalized frequency for each grid. If the grid size is too small, even subtle movements are to be recorded as transitions between grids, which results in higher entropy. Thus, it is necessary to set an appropriate grid size considering playing. We divided the sideline and endline into 10 and 15 grids, respectively, where each grid is approximately 100 cm ×100 cm in size. The length of 100 cm roughly corresponds to the minimum distance from fingertip to shoulder width in adult females [51]. It can be the minimum space required for various offensive actions, such as dribbling, passing, shooting, and staying in place. In this analysis, the court was divided into 150 grids in total, and entropy was calculated based on these grids. In the random walk condition, each offensive agent started from the actual initial position in the high expertise condition, and moved randomly one grid in the up, down, left, or right direction at each time frame; the entropy was calculated in the same manner.

### Procedures for variability in the distance with the key player

This study also conducted an additional fundamental analysis to supplement hypothesis (H1-2) and verify hypothesis (H3), explained in the Introduction section. As a reference, we focused on offensive #3 in the key role of intervention decision and adjustment (Fig 1) and calculated the coefficient of variation (CV) in the distance (cm) between offensive #3 and (A) each teammate and (B) each defensive player. Indices (A) and (B) were five combinations of the players. We aimed to capture an aspect of movement dynamics from a perspective different from entropy. Their distances were calculated for each time frame (0.05 s) in each trial and averaged through all the frames. Subsequently, the standard deviation was divided by the average to compute the CV. The CV is an index, which represents the degree of variability relative to the average. For calculating the CV and entropy, this analysis did not previously apply filtering to the raw position data to avoid excessive smoothing, considering the temporal resolution of 20 fps. No time window was applied because the mini-game had a short time limit of 15-s duration in principle.

A larger CV for (A) indicates relatively greater variability in the distance between offensive #3 in the key role of intervention decision and adjustment and each teammate. If the offensive team demonstrates coordination, such as “hand-off” and “pick and roll”, the distances may be relatively close. In contrast, the distances may be relatively far in another coordination, such as the key player running to a corner area (“deep corner”) and attempting a three-point shot, as illustrated in Fig 1. The complex characteristics may be related to the application of team strategies. The CV and entropy were calculated using MATLAB R2021b.

### Procedures for estimating the relationship between the condition and offensive movements

**Fig 5:**
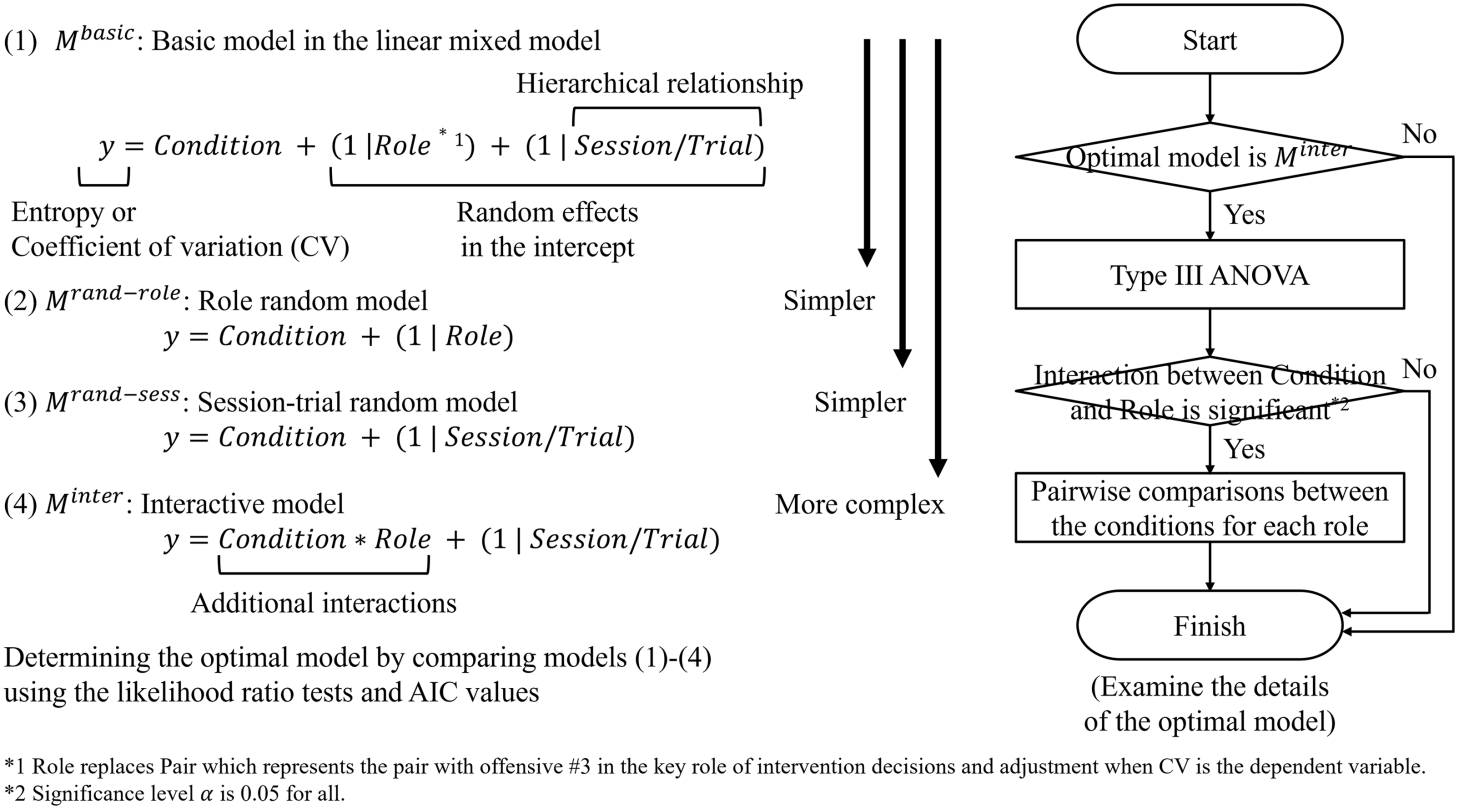
Analysis overview of the linear mixed model. The dependent variable is entropy, which represents the difficulty in anticipating offensive movements for the defensive team, or the coefficient of variation in the distance (cm) between offensive #3 in the key role of intervention decision and adjustment and each other player. The main independent variable is the categorical condition. The model descriptions are according to the coding of the R packages.

We conducted the statistical analysis according to the experimental design in the current and previous studies and the sample size [12]. Each mini-game was defined as one trial, and three sessions comprising seven trials per session were conducted, yielding a total of 21 trials. We prepared the four conditions: high expertise condition, low expertise before the tips condition, low expertise after the tips condition, and random walk condition through computer simulation. This analysis compared entropy, which represents the difficulty in offensive movements for the defensive team, with the four conditions, using a linear mixed model. The linear mixed model is a statistical method that estimates the relationship between dependent and independent variables by incorporating the fixed effects and random effects. The latter accounts for dependencies and variabilities in data arising from repeated measurements and hierarchical structures. We set four types of models and selected the best-fitting one. Then, we examined the relationship between the dependent and independent variables as explained by this optimal model; Fig 5 shows the details. The significance level α was set to 0.05 for all.

Entropy was used as the dependent variable, and the categorical condition was used as the independent variable. (1) The basic model included the role as the random effects in the intercept, as well as the session and trial. The session and trial have a hierarchical structure. As simpler models, (2) the role random model included the random effect of only the role in the intercept. (3) The session-trial random model included the random effect of only the session and trial in the intercept. As a more complex model, (4) the interactive model included the role and the interaction with the condition in the slope, in addition to the configuration of model (3). We compared these models using the likelihood ratio tests and Akaike Information Criterion (AIC) values, and selected as the optimal model based on the criteria of showing a significant improvement in the model fitting and the smallest AIC value. If model (4) was the optimal model, we conducted a Type III ANOVA to examine the significance of the interaction between the condition and role, as well as their main effects. If the interaction was significant, we further examined these details of the optimal model by pairwise comparisons of the entropy between the conditions for each role, in relation to the hypothesis testing. To conduct the multiple comparisons, p-values were adjusted using the Bonferroni method.

The coefficient of variation in the distance (cm) between offensive #3 in the key role of intervention decision and adjustment and each other player was analyzed in the same manner. The random walk condition data were not included for the CV analysis; the number of conditions was three. This statistical analysis was conducted using R-4.3.3, the lme4 1.1–37, lmerTest 3.1–3, emmeans 1.11.2–8, and dplyr 1.1.4 packages (S1 Dataset).

## Results

### Results of offensive team performance

Table 2 shows the offensive team performance in the high expertise, low expertise before the tips, and low expertise after the tips conditions. In the high expertise condition, the team achieved six wins and only one loss in each session, making of 18 wins and three losses. It indicated that the team consistently achieved the common goal of winning against the defensive team. A high Cohen’s Kappa coefficient was obtained for the trials in which the judgments were clearly determined (K = 0.767), indicating substantial agreement between the two evaluators. Disagreements were observed in only five out of the 21 trials, and all the final judgments were determined by the majority vote among the three evaluators.

**Table 2:** Offensive team performance in the high expertise, low expertise before the tips, and low expertise after the tips conditions.

Condition	Session	Win	Loss
High expertise	1	6	1
	2	6	1
	3	6	1
Low expertise before the tips	1	4	3
	2	4	3
	3	1	6
Low expertise after the tips	1	4	3
	2	6	1
	3	1	6

In the low expertise before the tips condition in the previous study [12], as the pretest, the offensive team recorded four wins and three losses in Sessions 1 and 2, and one win and six losses in Session 3, making nine wins and 12 losses. In the low expertise after the tips condition, as the posttest, the performance was four wins and three losses in Sessions 1, six wins and one loss in Session 2, and one win and six losses in Session 3, resulting in 11 wins and 10 losses. A high Cohen’s Kappa coefficient was also obtained (K = 0.731). Only 10 out of the 42 trials showed disagreement between the two evaluators. Nine trials were judged by the majority vote among the three evaluators; for the remaining trial, the first and second authors of the previous study discussed based on the evaluators’ comments and made a decision of the offensive team winning.

### Results of offensive movements making it difficult for the defensive team to anticipate

**Fig 6:**
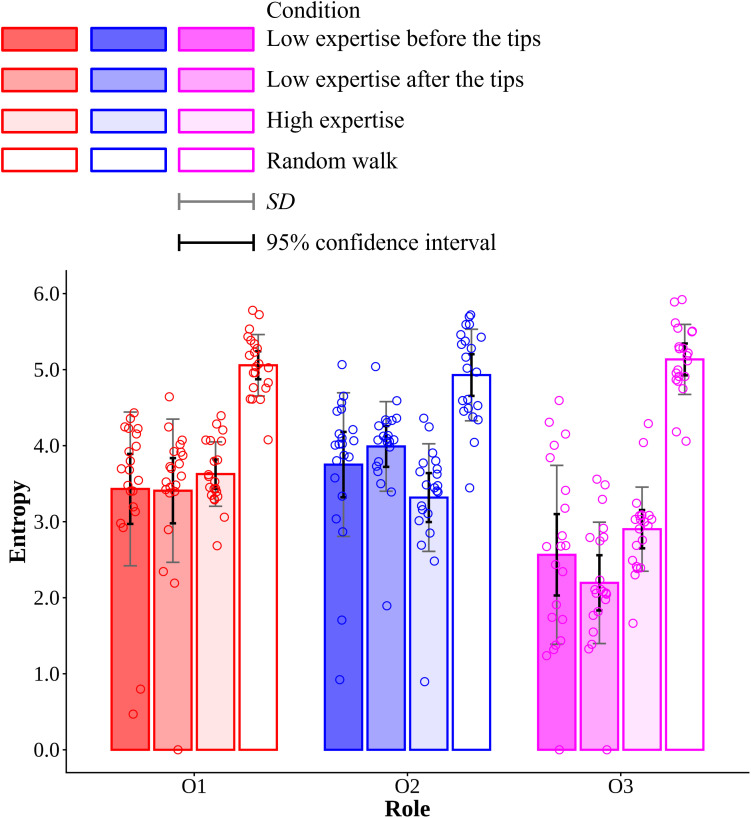
Entropy representing the difficulty in anticipating offensive movements for the defensive team. The horizontal and vertical axes present the role and entropy, respectively. For each role, the bars show the averages across the 21 trials for the four conditions. The gray error bars indicate the standard deviations, and the black error bars indicate 95% confidence intervals.

Fig 6 shows the entropy representing the difficulty in anticipating the offensive movements for the defensive team. The horizontal and vertical axes present the role and entropy, respectively.

For each role, the bars show the averages across the 21 trials for the four conditions. The gray error bars indicate the standard deviations, and the black error bars indicate 95% confidence intervals. This study estimated the relationship between the condition and entropy as the independent and dependent variables, using a linear mixed model at the significance level (α=0.05). Consequently, the likelihood ratio tests showed that the model fit significantly improved from model (3), which included the random effect of only the session and trial in the intercept, to model (1), which also included the random effect of the role in the intercept. Furthermore, the model fit significantly improved from model (1) to (4), which included the role and the interaction with the condition in the slope ($M^{rand-role}-M^{rand-sess}:\;\chi^{\text{2}}(\text{1})=\text{0}.\text{0}\text{0}\text{0},\;p=\text{1}.\text{0}\text{0}\text{0};\;M^{rand-sess}-M^{basic}:\;\chi^{\text{2}}(\text{1})=\text{3}\text{2}.\text{9}\text{1}\text{9},\;p<\text{0}.\text{0}\text{0}\text{1};\;M^{basic}-M^{inter}:\;\chi^{\text{2}}(\text{7})=\text{5}\text{4}.\text{2}\text{9}\text{8},\;p<\text{0}.\text{0}\text{0}\text{1}$). Model (4) had the smallest AIC value among the four models (${AIC}^{rand-role}=\text{6}\text{4}\text{0}.\text{0}\text{3},\;{AIC}^{rand-sess}=\text{6}\text{7}\text{2}.\text{6}\text{8},\;{AIC}^{basic}=\text{6}\text{4}\text{1}.\text{7}\text{6},{AIC}^{inter}=\text{6}\text{0}\text{1}.\text{4}\text{6})$ (Table 3). When estimating with the optimal model, the low expertise before the tips condition and role O1 were set as the baseline categories. The fixed effects of the intercept, the random walk condition, the key role O3 of intervention decision and adjustment, and the interaction between the random walk condition and role O3 were significant effects on the entropy across all the sessions and trials (${\hat{\beta }}^{inter}=\text{3}.\text{4}\text{3}\text{1},\;SE=\text{0}.\text{1}\text{7}\text{6},\;t(\text{4}\text{1}.\text{5}\text{0}\text{0})=\text{1}\text{9}.\text{4}\text{6}\text{6},\;p<\text{0}.\text{0}\text{0}\text{1};\;{\hat{\beta }}^{rand}=\text{1}.\text{6}\text{2}\text{6},\;SE=\text{0}.\text{2}\text{3}\text{2},\;t(\text{2}\text{2}\text{0}.\text{0}\text{0}\text{0})=\text{7}.\text{0}\text{1}\text{1},\;p<\text{0}.\text{0}\text{0}\text{1};\;{\hat{\beta }}^{O\text{3}}=-\text{0}.\text{8}\text{6}\text{7},\;SE=\text{0}.\text{2}\text{3}\text{2},\;t(\text{2}\text{2}\text{0}.\text{0}\text{0}\text{0})=-\text{3}.\text{7}\text{3}\text{6},\;p<\text{0}.\text{0}\text{0}\text{1};\;{\hat{\beta }}^{rand\times O\text{3}}=\text{0}.\text{9}\text{4}\text{4},\;SE=\text{0}.\text{3}\text{2}\text{8},\;t(\text{2}\text{2}\text{0}.\text{0}\text{0}\text{0})=\text{2}.\text{8}\text{7}\text{6},\;p=\text{0}.\text{0}\text{0}\text{4}$) (Table 4). Subsequently, we conducted a Type III ANOVA, revealing significant main effects of the condition and role as well as a significant interaction between them. These effect sizes were large (Condition: $F(\text{3},\;\text{2}\text{2}\text{0})=\text{9}\text{0}.\text{1}\text{9}\text{5},p<.\text{0}\text{0}\text{1},\;partial\;\eta^{\text{2}}=\text{0}.\text{5}\text{5}$; Role: $F(\text{2},\;\text{2}\text{2}\text{0})=\text{2}\text{7}.\text{6}\text{1}\text{0},p<.\text{0}\text{0}\text{1},\;partial\;\eta^{\text{2}}=\text{0}.\text{2}\text{0}$; Interaction: $F(\text{6},\;\text{2}\text{2}\text{0})=\text{7}.\text{6}\text{1}\text{7},p<.\text{0}\text{0}\text{1},\;partial\;\eta^{\text{2}}=\text{0}.\text{1}\text{7}$). Thus, for the optimal model, Bonferroni-corrected pairwise comparisons of the entropy between the conditions for each role were conducted in relation to the hypothesis testing. The results showed that the entropy in the random walk condition was significantly higher than that in the other conditions for all roles (ps<0.001). For role O2, the entropy in the high expertise condition was significantly lower than that in the low expertise after the tips condition (p=0.025). Notably, for role O3, the entropy in the high expertise condition was significantly higher than that in the low expertise after the tips condition (p=0.016) (Table 5). The random effects for the intercept showed that the variance between the sessions was 0.008 and the variance between the trials in the sessions was 0.033, indicating that the former was relatively small. The residual variance was 0.565.

**Table 3:** Likelihood ratio tests and AIC values using the linear mixed model for estimating the relationship between the condition and entropy. The entropy represents the difficulty in anticipating offensive movements for the defensive team. The values in the optimal model are shown in bold. The detailed specifications of each model are shown in Fig 5.

Model	AIC	$\chi^{\text{2}}$	𝐝𝐟	𝐩-value
$M^{rand-role}$	640.03	–	–	–
$M^{rand-sess}$	672.68	0.000	1	1.000
$M^{basic}$	641.76	32.919	1	< 0.001
$M^{inter}$	**601.46**	**54.298**	**7**	**< 0.001**

**Table 4:** Optimal model estimating the relationship between the condition and entropy which represents the difficulty in anticipating offensive movements for the defensive team. The values of the fixed effects whose *p*-values are below the significance level (α = 0.05) are shown in bold. The low expertise before the tips condition and role O1 are set as the baseline categories.

Fixed effects	$\hat{\beta }$	SE	𝐝𝐟	𝐭-value	𝐩-value
Intercept	**3.431**	**0.176**	**41.500**	**19.466**	**< 0.001**
Low expertise after the tips condition	−0.023	0.232	220.000	−0.101	0.919
High expertise condition	0.196	0.232	220.000	0.843	0.400
Random walk condition	**1.626**	**0.232**	**220.000**	**7.011**	**< 0.001**
Role O2	0.320	0.232	220.000	1.379	0.169
Role O3	**−0.867**	**0.232**	**220.000**	**−3.736**	**< 0.001**
Low expertise after the tips condition × Role O2	0.262	0.328	220.000	0.800	0.425
High expertise condition × Role O2	−0.629	0.328	220.000	−1.918	0.056
Random walk condition × Role O2	−0.448	0.328	220.000	−1.367	0.173
Low expertise after the tips condition × Role O3	−0.345	0.328	220.000	−1.053	0.294
High expertise condition × Role O3	0.142	0.328	220.000	0.432	0.666
Random walk condition × Role O3	**0.944**	**0.328**	**220.000**	**2.876**	**0.004**
Random effects	Variance				
Session/Trial’s intercept	0.033				
Session’s Intercept	0.008				
Residual	0.565				

**Table 5:** Pairwise comparisons of the entropy in the optimal model which represents the difficulty in anticipating offensive movements for the defensive team between the conditions for each role. The values of the fixed effects whose p-values are below the significance level (α = 0.05) are shown in bold.

Role	Comparison	$\hat{\beta }$	SE	𝐝𝐟	𝐭-value	𝐩-value
O1	Low expertise before the tips condition vs. Low expertise after the tips condition	0.024	0.232	220.000	0.101	1.000
	Low expertise before the tips condition vs. High expertise condition	−0.196	0.232	220.000	−0.843	1.000
	Low expertise before the tips condition vs. Random walk condition	**−1.627**	**0.232**	**220.000**	**−7.011**	**< 0.001**
	Low expertise after the tips condition vs. High expertise condition	−0.219	0.232	220.000	−0.944	1.000
	Low expertise after the tips condition vs. Random walk condition	**−1.650**	**0.232**	**220.000**	**−7.112**	**< 0.001**
	High expertise condition vs. Random walk condition	**−1.431**	**0.232**	**220.000**	**−6.168**	**< 0.001**
O2	Low expertise before the tips condition vs. Low expertise after the tips condition	−0.239	0.232	220.000	−1.030	1.000
	Low expertise before the tips condition vs. High expertise condition	0.434	0.232	220.000	1.869	0.377
	Low expertise before the tips condition vs. Random walk condition	**−1.178**	**0.232**	**220.000**	**−5.078**	**< 0.001**
	Low expertise after the tips condition vs. High expertise condition	**0.673**	**0.232**	**220.000**	**2.899**	**0.025**
	Low expertise after the tips condition vs. Random walk condition	**−0.939**	**0.232**	**220.000**	**−4.049**	**< 0.001**
	High expertise condition vs. Random walk condition	**−1.612**	**0.232**	**220.000**	**−6.948**	**< 0.001**
O3	Low expertise before the tips condition vs. Low expertise after the tips condition	0.369	0.232	220.000	1.590	0.680
	Low expertise before the tips condition vs. High expertise condition	−0.337	0.232	220.000	−1.454	0.884
	Low expertise before the tips condition vs. Random walk condition	**−2.570**	**0.232**	**220.000**	**−11.079**	**< 0.001**
	Low expertise after the tips condition vs. High expertise condition	**−0.706**	**0.232**	**220.000**	**−3.044**	**0.016**
	Low expertise after the tips condition vs. Random walk condition	**−2.939**	**0.232**	**220.000**	**−12.668**	**< 0.001**
	High expertise condition vs. Random walk condition	**−2.233**	**0.232**	**220.000**	**−9.625**	**< 0.001**

### Results of variability in the distance with the key player

**Fig 7:**
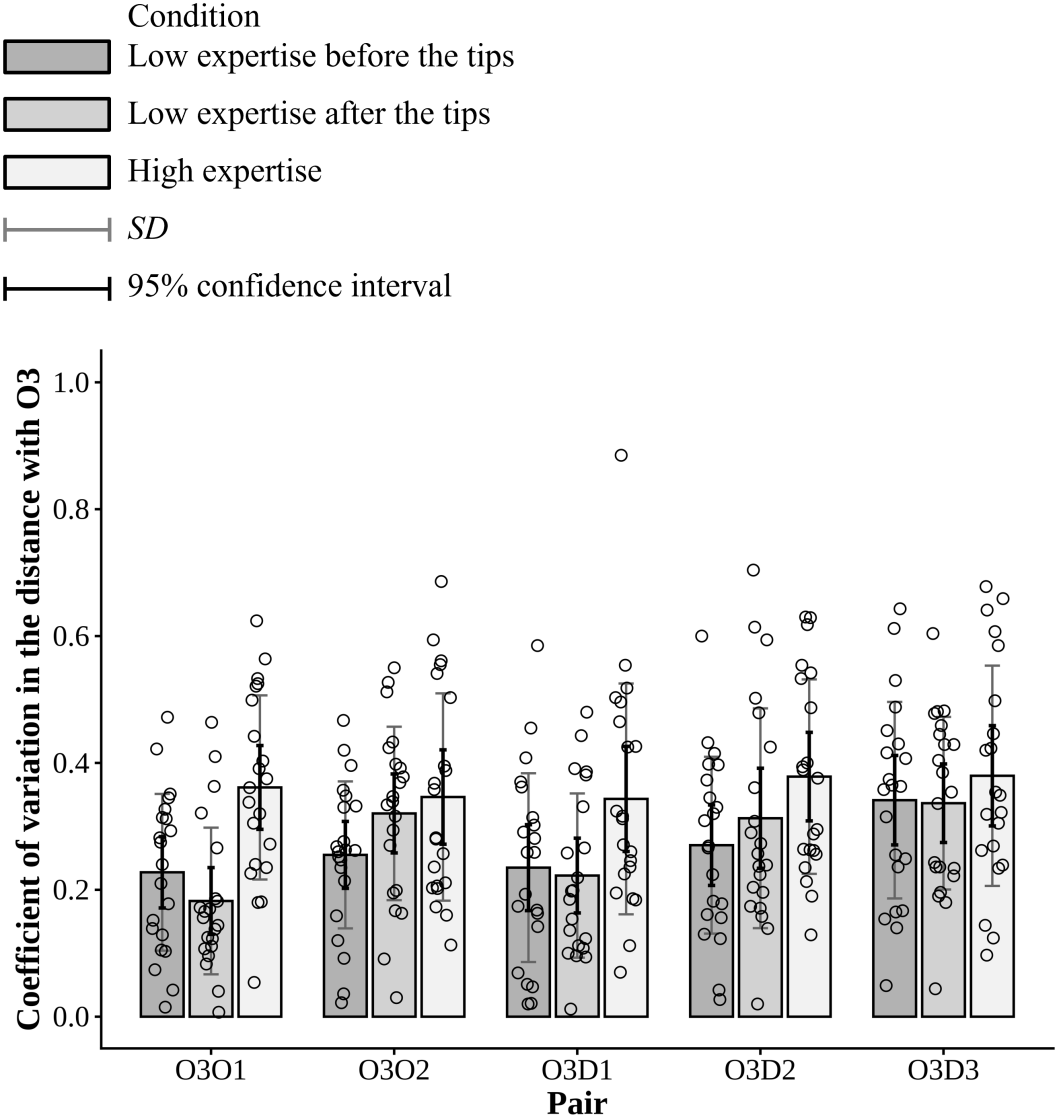
Coefficient of variation (CV) representing the variability in the distance (cm) between offensive #3 in the key role of intervention decision and adjustment and each other player. The horizontal and vertical axes represent the pair with offensive #3 and CV, respectively. For each pair, the bars show the averages across the 21 trials for the three expertise conditions. The gray error bars indicate the standard deviations, and the black error bars indicate 95% confidence intervals.

Fig 7 shows the coefficient of variation (CV) representing the variability in the distance (cm) between offensive #3 in the key role of intervention decision and adjustment and each other player. The horizontal and vertical axes present the pair with offensive #3 and CV, summarized similar to the entropy.

This study estimated the relationship between the condition and CV as the independent and dependent variables, using a linear mixed model. Consequently, the likelihood ratio tests showed that the model fit significantly improved from model (2), which included the random effect of only the pair with offensive #3 in the intercept, to model (3), which included the random effect of only the session and trial in the intercept. The model fit significantly improved from model (3) to model (1), which included the random effects of both pair and session and trial in the intercept. Furthermore, the model fit significantly improved from model (1) to (4), which included the pair and the interaction with the condition in the slope ($M^{rand-role}-M^{rand-sess}:\;\chi^{\text{2}}(\text{1})=\text{26}.\text{513},\;p<\text{0}.\text{0}\text{0}\text{1};\;M^{rand-sess}-M^{basic}:\;\chi^{\text{2}}(\text{1})=\text{9}.\text{471},\;p=\text{0}.\text{0}\text{0}\text{2};\;M^{basic}-M^{inter}:\;\chi^{\text{2}}(\text{1}\text{1})=\text{22}.\text{682},\;p=\text{0}.\text{0}\text{2}\text{0}$). Model (4) had the smallest AIC values among the four models (${AIC}^{rand-role}=-\text{2}\text{8}\text{1}.\text{8}\text{3},\;{AIC}^{rand-sess}=-\text{3}\text{0}\text{6}.\text{3}\text{4},\;{AIC}^{basic}=-\text{3}\text{1}\text{3}.\text{8}\text{1},{AIC}^{inter}=-\text{3}\text{1}\text{4}.\text{4}\text{9})$ (Table 6). When estimating with the optimal model, the low expertise before the tips condition and the pair of offensive #3 and offensive #1 were set as the baseline categories. The fixed effects of the intercept, the high expertise condition, and the pair of offensive #1 and defensive #3 were significant effects on the CV across all the sessions and trials (${\hat{\beta }}^{inter}=\text{0}.\text{2}\text{2}\text{8}\;,\;SE=\text{0}.\text{0}\text{3}\text{3},\;t(\text{1}\text{9}\text{0}.\text{3}\text{6}\text{5})=\text{6}.\text{9}\text{0}\text{4},\;p<\text{0}.\text{0}\text{0}\text{1};\;{\hat{\beta }}^{high}=\text{0}.\text{1}\text{3}\text{4},\;SE=\text{0}.\text{0}\text{4}\text{2},\;t(\text{2}\text{8}\text{0}.\text{0}\text{0}\text{0})=\text{3}.\text{2}\text{1}\text{3},\;p=\text{0}.\text{0}\text{0}\text{1};\;{\hat{\beta }}^{O\text{3}D\text{3}}=\text{0}.\text{1}\text{1}\text{4}\;,\;SE=\text{0}.\text{0}\text{4}\text{2},\;t(\text{2}\text{8}\text{0}.\text{0}\text{0}\text{0})=\text{2}.\text{7}\text{3}\text{1},\;p=\text{0}.\text{0}\text{0}\text{7}$). Subsequently, we conducted a Type III ANOVA, revealing significant main effects of the condition and pair. However, the interaction between the condition and pair was not significant. All the effect sizes were not large (Condition: $F(\text{2},\;\text{2}\text{8}\text{0})=\text{1}\text{6}.\text{2}\text{0}\text{4},\;p<.\text{0}\text{0}\text{1},\;partial\;\eta^{\text{2}}=\text{0}.\text{1}\text{0};\text{ Pair}:\;F(\text{4},\;\text{2}\text{8}\text{0})=\text{5}.\text{3}\text{3}\text{4},\;p<.\text{0}\text{0}\text{1},\;partial\;\eta^{\text{2}}=\text{0}.\text{0}\text{7};\text{ Interaction}:\;F(\text{8},\;\text{2}\text{8}\text{0})=\text{1}.\text{3}\text{7}\text{8},\;p=.\text{2}\text{0}\text{6},\;partial\;\eta^{\text{2}}=\text{0}.\text{0}\text{4}$). In the optical model, Bonferroni-corrected pairwise comparisons of the CV between the conditions for each pair could not be conducted. Thus, the notable results indicated that as the fixed effect, the CVs in the distance between offensive #3 and each other player in the high expertise condition were significantly larger than those in the low expertise before the tips condition (${\hat{\beta }}^{high}=\text{0}.\text{1}\text{3}\text{4},\;SE=\text{0}.\text{0}\text{4}\text{2},\;t(\text{2}\text{8}\text{0}.\text{0}\text{0}\text{0})=\text{3}.\text{1}\text{2}\text{3},\;p=\text{0}.\text{0}\text{0}\text{1}$) (Table 7). The random effects for the intercept showed that the variance between the sessions was 0.000 and the variance between the trials in the sessions was 0.005, indicating that the former was relatively small. The residual variance was 0.018.

**Table 6:** Likelihood ratio tests and AIC values using the linear mixed model for estimating the relationship between the condition and the coefficient of variation (CV). The CV represents the variability in the distance (cm) between offensive #3 in the key role of intervention decision and adjustment and each other player. The values in the optimal model are shown in bold. The detailed specifications of each model are shown in Fig 5.

Model	AIC	$\chi^{\text{2}}$	𝐝𝐟	𝐩-value
$M^{rand-role}$	−281.83	–	–	–
$M^{rand-sess}$	−306.34	26.513	1	< 0.001
$M^{basic}$	−313.81	9.471	1	0.002
$M^{inter}$	**−314.49**	**22.682**	**11**	**0.020**

**Table 7:** Optimal model estimating the relationship between the condition and the coefficient of variation (CV). The CV represents the variability in the distance (cm) between offensive #3 in the key role of intervention decision and adjustment and each other player. The values of the fixed effects whose p-values are below the significance level (α = 0.05) are shown in bold. The low expertise before the tips condition and the pair of offensive #3 and offensive #1 are set as the baseline categories.

Fixed effects	$\hat{\beta }$	SE	𝐝𝐟	𝐭-value	𝐩-value
Intercept	**0.228**	**0.033**	**190.365**	**6.904**	**< 0.001**
Low expertise after the tips condition	−0.045	0.042	280.000	−1.087	0.278
High expertise condition	**0.134**	**0.042**	**280.000**	**3.213**	**0.001**
Pair O3-O2	0.027	0.042	280.000	0.659	0.511
Pair O3-D1	0.007	0.042	280.000	0.176	0.860
Pair O3-D2	0.043	0.042	280.000	1.026	0.306
Pair O3-D3	**0.114**	**0.042**	**280.000**	**2.731**	**0.007**
Low expertise after the tips condition × Pair O3-O2	0.111	0.059	280.000	1.878	0.061
High expertise condition × Pair O3-O2	−0.043	0.059	280.000	−0.722	0.471
Low expertise after the tips condition × Pair O3-D1	0.033	0.059	280.000	0.556	0.578
High expertise condition × Pair O3-D1	−0.025	0.059	280.000	−0.432	0.666
Low expertise after the tips condition × Pair O3-D2	0.088	0.059	280.000	1.490	0.137
High expertise condition × Pair O3-D2	−0.026	0.059	280.000	−0.436	0.663
Low expertise after the tips condition × Pair O3-D3	0.040	0.059	280.000	0.688	0.492
High expertise condition × Pair O3-D3	−0.095	0.059	280.000	−1.619	0.107
Random effects	Variance				
Session/Trial’s intercept	0.005				
Session’s intercept	0.000				
Residual	0.018				

In the Discussion section, the hypothesis testing was reported based on all the results.

## Discussion

This study compared the offensive movements in 3-on-3 basketball across the different expertise levels. We calculated entropy, which represents the difficulty in anticipating the movements for the defensive team. The results show that hypotheses (H-1) and (H-2) were supported: for the key role of intervention decision and adjustment, the entropy in the high expertise condition was significantly higher than that in the low expertise after the tips condition, and similar to that in the low expertise before the tips condition (Fig 6 and Table 5). Hypothesis (H2) was also supported because the entropy in the random walk condition was significantly higher than that in the other conditions for all roles. When compared with the observed movements, the differences from the random walk condition would be self-evident. Furthermore, hypothesis (H3) was supported: the coefficient of variation (CV), which represents the variability in the distance (cm) between offensive #3 in the key role and each other player, the CVs in the high expertise condition were significantly larger than those in the low expertise before the tips condition (Fig 7 and Table 7).

**Fig 8:**
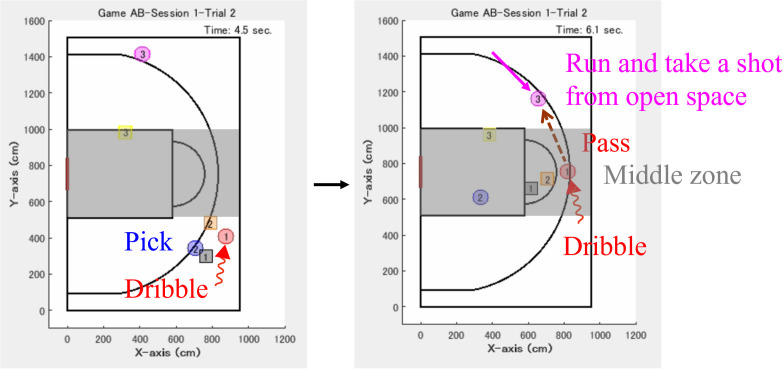
Example of team strategies observed in the high expertise condition. Trial 2 of Session 1 lasted about 8 s and resulted in a win for the offensive team. A solid arrow represents the movements of the off-ball player, zigzag arrows present those of the on-ball player, and a dotted arrow indicates the pass trajectory.

In team sports, it is particularly crucial for players to move in an organized manner while engaging in complex interactions that are difficult for the opponent to anticipate [29,52]. The movement dynamics at the expert level may be relatively complex, making it difficult for the defensive team to anticipate. Such a tendency may be observed in the offensive key role of intervention decision and adjustment. Fig 8 shows a snapshot visualizing the offensive movements using the time-series position data in Trial 2 of Session 1. Around 4.5 s after the start, as her own role, offensive #1 aimed to dribble from near the sideline toward the “middle zone” (central area by dividing the endline into three segments) and drew defensive #2. Concurrently, offensive #2 did screen play against defensive #1, who was marking offensive #1, thereby releasing the defensive pressure. This coordination created a favorable situation. Defensive #3, who was marking offensive #3, was positioned approximately 8 m away from offensive #1. This player had to shorten the distance with offensive #1 because of open space. Subsequently, around 6.1 s after the start, offensive #1 passed the ball to offensive #3 who ran toward the open space. Offensive #3 took a shot near the three-point line. The trial, which lasted about 8 s, resulted in a win for the offensive team. The complex characteristics may be related to the application of team strategies. As a reference, in our study in which the interview was conducted after the mini-game, the offensive team reported questions regarding crucial coordination [53] (S2 Note). The main results suggested that the team shared several strategies. Meanwhile, the entropy for offensive #2 in the high expertise condition showed the opposite results to that of offensive #3. It was significantly lower than that in the low expertise after the tips condition (Fig 6 and Table 5). This might be influenced by individual characteristics. According to the head coach (the third author), offensive #2 was taller, and the player blocked (picked) a defensive player by utilizing the physical characteristic.

The offensive team in the low expertise condition might establish a representative coordination pattern by the tips, such as the key player running to a corner area and attempting a three-point shot. However, the defensive team established an effective countermeasure during the posttest, as reported by the defensive players. The offensive team performance suddenly decreased at Session 3 in the low expertise after the tips condition (Table 2). Coordination established in the low expertise after the tips might be limited in flexibility according to situations [12]. Therefore, if the generalizability and validity of the findings can be fully ensured, these may be a practical suggestion for coaching and training, referring from the previous study on the low expertise condition. For example, a defensive player approaches to help, offensive #3 intervenes with the teammates and adjusts her own movement by checking the positions of the on-ball player and the defensive player, who marks offensive #3. Subsequently, offensive #3 runs (1–1) to a corner area or (1–2) to the goal (“Goal-cut”), receives a pass, and attempts an uncontested shot (“a lay-up shot”). Alternatively, (2) offensive #3 does not intervene with the teammates and stays in place to create open space for them. The on-ball player still dribbles and attempts a shot without passing (S3 Note). Thus, the findings may propose that coaching and training can facilitate the acquisition of diverse coordination based on several team strategies, making it difficult for the defensive team to anticipate.

This study may provide intriguing insight as a first step to understand the relationship between coordination, expertise, and the application of team strategies. Previous studies often analyzed movement dynamics at the expert level, including professional teams (e.g., [54,55]). In contrast, this study also focused on the low expertise condition. However, a lot of future research remains. In this study, the players in the high expertise and low expertise conditions were drawn from only a single affiliation, respectively. Furthermore, the indices in this analysis did not include contextual information, such as ball movement and action events (e.g., passing and dribbling). We did not directly examine which strategies the offensive team applied under what situations. The entropy and CV were the proxies that might be related to the application of team strategies, and the interpretations remain speculative. Therefore, conducting applied analysis that combines time-series action events with position data will be essential to address the problem in future studies. In addition, the mini-games were repeatedly conducted in this field experiment. We should request cooperation from more affiliated teams and conduct the field experiment designed to suit multiple game scenarios to verify the generalizability and validity of the findings.

## Conclusion

This study investigated the application of team strategies for dynamic coordination using 3-on-3 basketball across the different expertise levels. In this field experiment, the female players with higher expertise repeatedly engaged in the mini-games. We analyzed the difficulty in anticipating offensive movements for the defensive team, quantified as entropy. The results showed that the entropy for the key player in the high expertise condition was significantly higher than that in the low expertise after the tips condition, and similar to that in the low expertise before the tips condition. As a first step, our study suggests that the movement dynamics at the expert level may be relatively complex, making it difficult for the defensive team to anticipate, and related to the application of several team strategies. We also focused on the low expertise condition. Thus, if the generalizability and validity of the findings can be fully ensured, these may be a practical suggestion for coaching and training. However, these interpretations remain speculative, and there is much future research. To improve the generalizability and validity of the findings, we should request cooperation from more affiliated teams and conduct the field experiment designed to suit multiple game scenarios. Applied analysis that combines time-series action events (e.g., passing and dribbling) with position data should be conducted.

## Supporting information

S1 Note Details of experimental procedures. (PDF)

S2 Note Original findings different from the proceedings in the international conference. (PDF)

S3 Note Practical suggestion for coaching and training based on the findings. (PDF)

S1 Fig Example of practical suggestion for coaching and training based on the findings. (TIF)

S1 Table Results of the post-hoc questionnaire. (PDF)

S1 Dataset Dataset. (XLSX)
