# Supplementary material for: Applying team strategies for dynamic coordination: A comparative study of expertise using 3-on-3 basketball
Source: PLoS One. 2026 Feb 20;21(2):e0343077. doi: 10.1371/journal.pone.0343077 (PMC12923147; doi:10.1371/journal.pone.0343077)
Supplement: S1 Note — (PDF) [file pone.0343077.s001.pdf]

S1 Note. Details of experimental procedures

After the players repeatedly engaging in mini-games, we asked each player to look back and answer a post-hoc questionnaire for their offence as a reference. All the participants quantitatively evaluated the following questions on the degrees of (Q1) goal achievement, (Q2) sense of unity, (Q3) information sharing of crucial team coordination, (Q4) information sharing of crucial countermeasures against the opponent, (Q5) collective efficacy, and (Q6) game contribution. The players responded to Q1–Q4 using a 7-point Likert scale (0: Not well at all to 6: Very well). Meanwhile, they rated Q5 on 11-steps from −5 to +5 with 0 representing the baseline prior to this field experiment, and the values indicating negative or positive outcome. For Q6, each player rated their own contribution from 0 to 100 with 100 as the total contribution of the three players. These questions were referred from previous studies [1-3]. After the questionnaire, we conducted an interview with the offensive team (S2 Note).

S1 Table presents the results for each offensive player. Overall, a tendency toward positive responses was observed. In this field experiment, the offensive team achieved 18 wins and three losses in total, as explained in the Results of offensive team performance section. Therefore, high team performance was reflected in their subjective evaluations.

References

1. Kawazu K, Sugiyama Y, Nakasuga T. An investigation of the changing relationship between the collective efficacy and team performance of a sport team for different sporting event. Jpn J Sport Psychol. 2012;39(2):153–167. doi:10.4146/jjspopsy.2012-1120

2. Ichikawa J, Yamada M, Fujii K, Takeuchi Y. An exploratory study on coordinated role sharing during outdoor cooking. Jxiv [Preprint]. 2024. doi:10.51094/jxiv.590

3. Ichikawa J, Yamada M, Fujii K. Analyzing coordinated group behavior through role-sharing: a pilot study in female 3-on-3 basketball with practical application. Front Sports Act Living. 2025;7:1513982. doi:10.3389/fspor.2025.1513982
