# Supplementary material for: Applying team strategies for dynamic coordination: A comparative study of expertise using 3-on-3 basketball
Source: PLoS One. 2026 Feb 20;21(2):e0343077. doi: 10.1371/journal.pone.0343077 (PMC12923147; doi:10.1371/journal.pone.0343077)
Supplement: S2 Note — (PDF) [file pone.0343077.s002.pdf]

S2 Note. Original findings different from the proceedings in the international conference

At the international conference, we reported the results of a post-hoc interview with the offensive team after the mini-game [1]. This manuscript in the proceedings was published following a light peer-review process. As a reference and preliminary investigation, the previous study explored the extent to which information was shared among the players for crucial coordination. The results showed that while the team shared several strategies, these specific details were not fully shared; instead, the players mutually adjusted depending on the teammates' behaviors and situations. Meanwhile, this study recorded and analyzed their dynamic behaviors using tracking position data to examine the application of team strategies. We compared the movement dynamics across the different expertise levels. The purpose and analysis approach of this study significantly differed from those of the previous study. The findings of this study provide insight into implicit coordination that is difficult to obtain from the verbal reports. Thus, this study complements and extends the previous findings.

We sufficiently provide the original findings here. The manuscript has been already revised by more than 30%, as required by the consent to publish agreement from

the conference. As mentioned above, this is because the purpose and analysis approach of this study greatly differed from those of the previous study.

Reference

1. Ichikawa J, Yamada M, Iwaihara Y, Ichinose G. Conceptual model of information sharing and interaction in role-sharing for high team performance through a pilot study. In: Proceedings of the 27th International Conference on Human-Computer Interaction. Cham: Springer; 2025. p. 323–331.
