# Supplementary material for: Applying team strategies for dynamic coordination: A comparative study of expertise using 3-on-3 basketball
Source: PLoS One. 2026 Feb 20;21(2):e0343077. doi: 10.1371/journal.pone.0343077 (PMC12923147; doi:10.1371/journal.pone.0343077)
Supplement: S3 Note — (PDF) [file pone.0343077.s003.pdf]

S3 Note. Practical suggestion for coaching and training based on the findings

As explained in the manuscript, if the generalizability and validity of our findings can be fully ensured, these may develop a practical suggestion for coaching and training, referring from the previous study on the low expertise condition [1]. S1 Fig. shows an example. When a defensive player approaches to help, offensive #3 intervenes with the teammates and adjusts her own movement by checking the positions of the on-ball player and the defensive player, who marks offensive #3. Subsequently, offensive #3 runs (1-1) to a corner area or (1-2) to the goal ("Goal-cut"), receives a pass, and attempts an uncontested shot ("a lay-up shot"). Alternatively, (2) offensive #3 does not intervene with the teammates and stays in place to create open space for them. The on-ball player still dribbles and attempts a shot without passing. Thus, the findings may propose that coaching and training can facilitate the acquisition of diverse coordination based on several team strategies, making it difficult for the defensive team to anticipate.

Reference

1. Ichikawa J, Yamada M, Fujii K. Analyzing coordinated group behavior through role-sharing: a pilot study in female 3-on-3 basketball with practical application. Front Sports Act Living. 2025;7:1513982. doi:10.3389/fspor.2025.1513982

S1 Fig. Example of practical suggestion for coaching and training based on the findings. It is necessary to note that the generalizability and validity of the findings are fully ensured in future studies. Solid arrows represent the movements of the off-ball player, zigzag arrows present those of the on-ball player, and a dotted arrow indicates the pass trajectory.
