# Supplementary figures and images for: Applying team strategies for dynamic coordination: A comparative study of expertise using 3-on-3 basketball
Source: PLoS One. 2026 Feb 20;21(2):e0343077. doi: 10.1371/journal.pone.0343077 (PMC12923147; doi:10.1371/journal.pone.0343077)

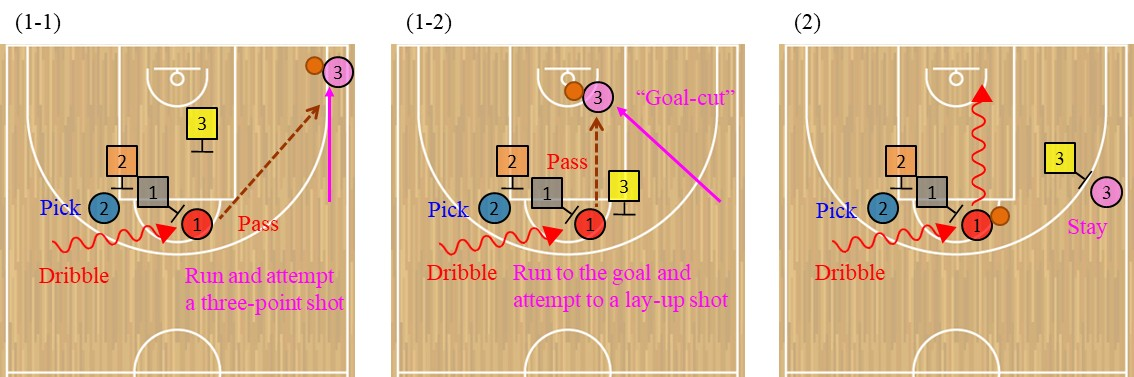

Supplement: S1 Fig — (TIF) [file pone.0343077.s004.tif]
