# Supplementary material for: Applying team strategies for dynamic coordination: A comparative study of expertise using 3-on-3 basketball
Source: PLoS One. 2026 Feb 20;21(2):e0343077. doi: 10.1371/journal.pone.0343077 (PMC12923147; doi:10.1371/journal.pone.0343077)
Supplement: S1 Table — (PDF) [file pone.0343077.s005.pdf]

S1 Table. Results of the post-hoc questionnaire

Question	Offensive player		
	#1	#2	#3
Q1 Goal achievement	4	5	6
Q2 Sense of unity	4	6	6
Q3 Information sharing of team crucial coordination	4	6	6
Q4 Information sharing of crucial countermeasures against the opponent	4	4	3
Q5 Collective efficacy	4	3	3
Q6 Game contribution	50	35	80

Note. Q1–Q4 were rated on a 7-point Likert scale (0: Not well at all to 6: Very well). Q5 responses were indicated by 11-steps from −5 to +5 with 0 representing the baseline prior to this field experiment, and the values indicating negative or positive outcome. Q6 responses ranged from 0 to 100 with 100 as the total contribution of the three players.
